# PQQ Dietary Supplementation Prevents Alkylating Agent-Induced Ovarian Dysfunction in Mice

**DOI:** 10.3389/fendo.2022.781404

**Published:** 2022-03-07

**Authors:** Xiuliang Dai, Xiangjiao Yi, Yufeng Wang, Wei Xia, Jianguo Tao, Jun Wu, Dengshun Miao, Li Chen

**Affiliations:** ^1^Department of Reproductive Medicine Center, The Affiliated Changzhou Maternal and Child Health Care Hospital of Nanjing Medical University, Changzhou, China; ^2^Institute of Orthopaedics and Traumatology, The First Affiliated Hospital of Zhejiang Chinese Medical University (Zhejiang Provincial Hospital of Traditional Chinese Medicine), Hangzhou, China; ^3^Department of Pathology, The Affiliated Changzhou Maternal and Child Health Care Hospital of Nanjing Medical University, Changzhou, China; ^4^Disease & Population (DaP) Geninfo Lab, School of Life Sciences, Westlake University, Hangzhou, China; ^5^Institute of Basic Medical Sciences, Westlake Institute for Advanced Study, Hangzhou, China; ^6^The Research Center for Bone and Stem Cells, Department of Anatomy, Histology and Embryology, Nanjing Medical University, Nanjing, China; ^7^The Research Center for Aging, Affiliated Friendship Plastic Surgery Hospital of Nanjing Medical University, Nanjing Medical University, Nanjing, China

**Keywords:** pyrroloquinoline quinine, alkylating agents, ovarian dysfunction, protection, ovarian aging

## Abstract

Alkylating agents (AAs) that are commonly used for cancer therapy cause great damage to the ovary. Pyrroloquinoline-quinine (PQQ), which was initially identified as a redox cofactor for bacterial dehydrogenases, has been demonstrated to benefit the fertility of females. The aim of this study was to investigate whether PQQ dietary supplementation plays a protective role against alkylating agent-induced ovarian dysfunction. A single dose of busulphan (20 mg/kg) and cyclophosphamide (CTX, 120 mg/kg) were used to establish a mouse model of ovarian dysfunction. Feed containing PQQNa_2_ (5 mg/kg) was provided starting 1 week before the establishment of the mouse model until the date of sacrifice. One month later, estrous cycle period of mice were examined and recorded for consecutive 30 days. Three months later, some mice were mated with fertile male mice for fertility test. The remaining mice were sacrificed to collect serum samples and ovaries. One day before sacrifice, some mice received a single injection of BrdU to label proliferating cells. Serum samples were used for test hormonal levels. Ovaries were weighted and used to detect follicle counts, cell proliferation, cell apoptosis and cell senescence. In addition, the levels of inflammation, oxidative damage and Pgc1α expression were detected in ovaries. Results showed that PQQ treatment increased the ovarian weight and size, partially normalized the disrupted estrous cycle period and prevented the loss of follicles of mice treated with AAs. More importantly, we found that PQQ treatment significantly increased the pregnancy rate and litter size per delivery of mice treated with AAs. The protective effects of PQQ appeared to be directly mediated by promoting cell proliferation of granulosa, and inhibiting cell apoptosis of granulosa and cell senescence of ovarian stromal cells. The underlying mechanisms may attribute to the anti-oxidative stress, anti-inflammation and pro-mitochondria biogenesis effects of PQQ.Our study highlights the therapeutic potential of PQQ against ovarian dysfunction caused by alkylating agents.

## Introduction

Alkylating agents (AAs) which have been widely used in the treatment of cancer also attack normal organs, and result in organ dysfunction (1, 2). Of these organs, the ovary is extremely sensitive to AAs (3). The ovarian dysfunction caused by AAs is mainly characterized by elevated FSH (Follicle-stimulating hormone) and LH (Luteinizing hormone) levels, decreased E2 (estradiol) production, disrupted estrous cycle periods, depleted ovarian reserves, ovarian stroma fibrosis and ovarian vessel damages (4). It has been demonstrated that treatment with AAs in childhood is strongly associated with the occurrence of premature ovarian insufficiency (POI) (5). Animal studies also demonstrate the serious toxic effect of AAs on the ovary, and AAs, are commonly used to establish mouse models of ovarian dysfunction (6–8). Busulphan (BUL) and cyclophosphamide (CTX) are two kinds of AAs which are frequently used in cancer therapy. Use of either BUL or CTX alone can damage the ovary. Sometimes, these two drugs will be used in combination. Jiang et al. showed that only a single injection of BUL and CTX can cause accelerated follicles depletion in mice, mimicking the process of ovarian aging (6). This mouse model has been widely adopted as a mouse model of POI for study (9–11).

The precise mechanisms underlying the ovarian damage caused by AAs are complicated and remain unclear. First wave of damage is directly caused by the toxicity of AAs. AAs can induce inter-and intra-strand DNA crosslinking that leads to apoptosis and cell death (4). Extensive cell death may create a specific microenvironment within ovary characterized by high level of oxidative stress and inflammation. That is consistent with the finding that ovary damage caused by AAs could be alleviated by inhibiting oxidative stress and inflammation (12). In addition, a recent study showed that mitochondrial protection could be a strategy against alkylating agent-induced ovarian damage, indicating a role of mitochondra impairment in mediating the ovarian toxicity of alkylating agents (13). Therefore, AAs damage ovary *via* multiple mechanisms. Finding a safe agent that can protect the ovary from being damaged by alkylating agents by targeting multiple pathways is an important issue to be addressed.

Pyrroloquinoline-quinine (PQQ) was first shown to function as a redox cofactor for dehydrogenases in bacteria (14). Later studies revealed that PQQ may also act as a cofactor for enzymes in eukaryote, such as PQQ-dependent sugar oxidoreductase activity in mushrooms and PQQ-mediated regulation of lactate dehydrogenase activity in mammals (15, 16). Although PQQ cannot be synthesized in mammals, it is available in foods such as milk, vegetables and meat (17, 18). Lack of PQQ in food resulted in developmental abnormalities in mice, including decreased fertility of female mice and survival of their offspring, growth retardation and impaired immune response (19, 20). In contrast, supplementation with PQQ was reported to alleviate the progression of multiple diseases in mice (21–29). More importantly, it has been reported that PQQ supplementation increased the reproductive performance of SD rats, and protected the ovaries of mice from ischemia-reperfusion injury (30, 31). In addition, a recent study showed that PQQ supplementation increased the pups per delivery of C57BL 6 background mice (32).

The mechanisms underlying the beneficial effects of PQQ on health may depend on its several biological effects including its anti-oxidative stress, anti-inflammation, and pro-mitochondria biogenesis effects. PQQ is known as the strongest water-soluble antioxidant (the singlet molecular oxygen-quenching activity of PQQ was approximately 6.3-times higher than that of vitamin C) (33). Uptake of PQQ has been shown to decrease the levels of plasma C-reactive protein and interleukin (IL)-6 and promote the biogenesis of mitochondria in humans (34, 35). In addition, dietary supplements containing PQQ have been available in the United States since 2009 (36). However, it remains unknown whether PQQ supplementation alleviates the ovary dysfunction caused by AAs. Therefore, we proposed that PQQ may be an ideal agent to alleviate damage to the ovary caused by AAs.

In the present study, we aimed to test the effect of PQQ supplementation on alkylating agent-induced ovarian dysfunction in mice. The main measures included fertility, hormone levels, estrous cycle period, follicle counts, ovarian cell proliferation/apoptosis/senescence, oxidative damage, inflammation, and mitochondrial marker in the ovary.

## Materials and Methods

### Animals, Establishment of Ovarian Dysfunction Mouse Model and Treatment

All the mice (C57BL/6 background) were maintained in a specific pathogen free (SPF) animal laboratory in Changzhou Kawensi Laboratory Animal Centre. A total of 60 female mice (8-week) were randomly divided into 3 groups, the control group (CON), ovary dysfunction group (O) and ovary dysfunction with PQQ supplementation group (OP). To establish an ovarian dysfunction mouse model, female mice aged 8 weeks received a single dose of BUL (Sigma, China, 20 mg/kg) and CTX (Selleck, China, 120 mg/kg) by intraperitoneal injection. The reagents were dissolved in DMSO, and further diluted in saline. The injected volume was 10 µL/g (body weight). The control mice were injected with an equal amount of solvent. For the mice in the OP group, feed containing PQQNa_2_ (5 mg/kg) was provided starting 1 week before the establishment of the mouse model until the date of sacrifice. For the mice in the CON and O groups, feed without PQQ was provided. Three months later, the mice were mated with 10-week old fertile male mice or sacrificed for further analysis. Twenty-four hours before sacrifice, 3 mice in each group were injected with BrdU (Sigma, 50 mg/kg). All the animal procedures and experiments were approved by the ethics committee of Changzhou Maternal and Healthy Care Hospital and Nanjing Medical University.

### Fertility Test

Three months after a single injection of BUL and CTX, seven female mice in each group were mated with 10-week old fertile male mice (C57BL/6 background) for 2 month. The fertility of the male mice was confirmed by mating experiment. The 8 week old male mice mated with 3 adult females, if one of the female mice got pregnancy, we considered that the male mice were fertile. After a confirmation of fertility of male mice, one female mouse was mated with one male mouse in per cage. The pregnancy, litter size of each mouse and the days from mating to delivery were recorded.

### Detection of the Estrous Cycle Period

Six mice for each group were used for monitoring estrous cycles starting 1 month after a single injection of BUL and CTX for consecutive 30 days. The estrous cycle was determined by analyses of vaginal smears. Ten microliters of saline was injected into the vagina and collected by suction 3 times. Then, the saline was smeared on a slide. After air drying, the smear was fixed with 95% ethanol. Then, HE staining was performed to analyze the estrous cycle period. The experiment of vaginal smear was conducted between 9:00 and 10:00 am every day.

### AMH, FSH and E2 Assays

Mice were sacrificed at 3 months after a single injection of BUL and CTX. Serum samples from mice in the diestrus phase were chosen to measure serum hormone levels. AMH, FSH and E2 were determined by ELISA, and anti-AMH (RJ-17440), anti-FSH (RJ-17024) and anti-E2 (RJ-17014) ELISA kits were used. All the ELISA kits were purchased from Shanghai Renjie Biological Company. All the procedures were performed strictly according to the instructions provided by the manufacturer.

### Follicle Counts

The paraffin-embedded ovaries were serially sectioned at 30 µm intervals, starting from the appearance of ovary tissue in the section. The thickness of the section was 5 µm. A total of 30 sections for each ovary were mounted on the slides. HE staining was performed and photos were taken with a light microscope. The numbers of the various types of follicles, including primordial follicles, primary follicles, secondary follicles, mature follicles and degenerative follicles, were counted. The criteria for the classification of follicles were previously described by Umehara et al. (37).

### Immunohistochemistry

Following dewaxing, hydration, antigen-retrieval with citrate buffer (0.01 M) by the high pression method, inactivation of endogenous peroxidase by 3% H_2_O_2_, and blocking of nonspecific binding sites by 10% donkey serum, the ovarian sections were incubated with primary antibodies and stored at 4°C overnight. The slides were washed with PBS 3 times (5 min/wash) to remove the unbound primary antibodies, and the sections were incubated with HRP-labeled secondary antibodies at room temperature for 1 hour. Following removal of the unbound secondary antibodies, positive staining of the sections was visualized by staining with a DAB solution (Beyotime Biotechnology). For BrdU immunohistochemistry, additional steps were needed. Concentrated hydrochloric acid was diluted in water at a ratio of 1:5 (v:v). The sections were incubated with diluted hydrochloric acid for 30 min at RT. Then, a boric acid solution was directly added to the sections for 20 min at RT. The primary antibodies for IHC in the present study included anti-p65 antibody (GB11142, Servicebio, China), anti-BrdU antibody (B2531,Millipore, China), anti-β-gal antibody (#2372, Cell Signaling Technology, China) and anti-8-OHdG antibody (sc-393871, Santa Cruz, China). The secondary antibodies included HRP-labeled goat anti-mouse and anti-rabbit antibodies (A0208 and A0216, Beyotime Biotechnology, China). Ovarian section without incubating with primary antibody was set as a negative control (**Supplementary Figure 1**).

### q-PCR

Total RNA was extracted by the TRIzol reagent. Single-strand DNA was synthesized by a reverse transcription kit (Vazyme, China). To detect the expression of the mRNA levels, real-time fluorescence quantitative PCR (SYBR green) was performed. The cycle conditions were as follows: a. denaturation at 95°C for 5 min. b. reaction cycles: 40 cycles of 95°C for 10 s and 60°C for 30 s. c. melt curve: 95°C for 15 s, 60°C for 60 s and 95°C for 15 s. The sequences of the primers specific for each mRNA detected in this study are listed in **Supplementary Table 1**. The relative expression of the mRNA levels was calculated by using the 2^△△Ct^ method.

### TUNEL Assay

The TUNEL assay was performed using a TUNEL assay kit (Roche, Switzerland). Ovarian sections were used for detection. All the procedures were performed strictly according to the instructions provided by the manufacturer.

### MDA Assay

The ovaries were removed and homogenized in cold normal saline. The homogenates were centrifuged at 2500 r/min for 10 min. The supernatants were collected for preparation. The MDA kit was purchased from Nangjing Jiancheng Bioengenineering Institute. All the procedures were performed strictly according to the instructions provided by the manufacturer.

### Statistical Analysis

All the values are presented as mean ± SD. P<0.05 was considered statistically significant. One-way ANOVA followed by *post-hoc* Tukey’s honestly significant difference test was used to compare data among 3 groups. SPSS software (ver. 18.0; SPSS Inc., Chicago, IL, USA) was used for the statistical analyses.

## Results

### The Effect of PQQ Treatment on the Fertility and Ovarian Function of Female Mice Treated With CTX in Combination With BUL (CTX/BUL)

**Figure 1:**
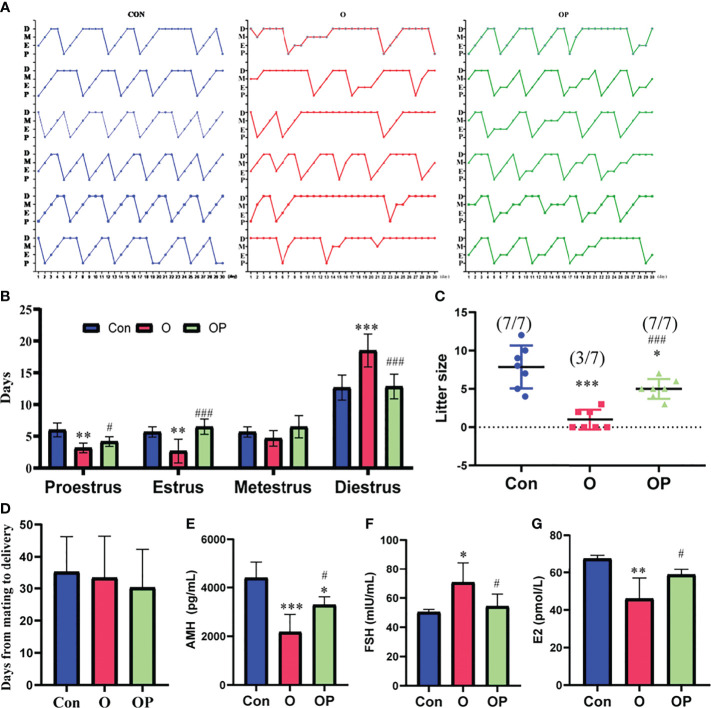
PQQ treatment improved the fertility and ovarian function of female mice treated with CTX in combination with BUL (CTX/BUL). **(A)** Record of estrous cycles of mice for each day, n = 6 mice in each group. **(B)** Estrous cycle period distribution for 30 consecutive days, n = 6 mice in each group. **(C)** Fertility test of female mice, n = 7 mice in each group. (pregnancy mice/total mice). **(D)** Days from mating to delivery, n = 7 mice in each group. ELISA analysis of serum **(E)** AMH, **(F)** FSH and **(G)** E2 levels, n = 4 samples in group Con, n=7 samples in group O, and n=5 samples in group OP. D, diestrus; M, metestrus; E, estrus; P, proestrus; AMH, anti-Mullerian hormone; FSH, follicle-stimulating hormone; E2, estradiol; PQQ, pyrroloquinoline; CTX, cyclophosphamide; BUL, busulfan. Compared with Con: *P < 0.05; **P < 0.01; ***P < 0.001; compared with O: ^#^P < 0.05; ^###^P < 0.001.

To determine whether estrus cycles in the mice in each group were normal, we detected estrous cycle period for each mice. Our results showed that the mice in group O showed irregular estrus cycles compared to the mice in group Control and group OP (**Figure 1A**). Statistical analysis revealed that the mice in group O had extended diestrus duration, and shortened proestrus and estrus durations, compared to the mice in group Control and group OP (**Figure 1B**). Subsequently, we tested the fertility of the mice in each group. We showed that the rate of pregnancy was significantly decreased in group O (3/7) compared to that in group Control (7/7) and group OP (7/7) (**Figure 1C**). Consistently, the litter size in group O was significantly reduced compared to group Control and group OP (**Figure 1C**). Group OP also showed a significantly reduced litter size compared to group Control (**Figure 1C**). However, the days from mating to delivery were similar among groups (**Figure 1D**). To further determine the ovarian function, we tested the related hormone levels. We found that the level of serum AMH which is a maker of ovarian reserve was significantly reduced in group O, compared to group Control and group OP (**Figure 1E**). The level of serum AMH in group OP was also decreased significantly compared to group Control (**Figure 1E**). In addition, we found that the level of serum FSH was significantly increased while the level of E2 was significantly decreased in group O, compared to group Control and group OP (**Figures 1F, G**). These results indicated that PQQ treatment partially protected ovarian function and fertility of the mice challenged by CTX/BUL.

### The Effect of PQQ Treatment on Follicle Counts of Female Mice Treated With CTX/BUL

**Figure 2:**
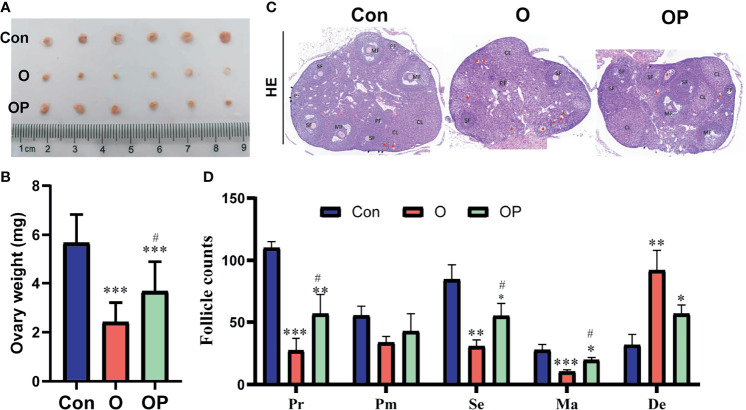
PQQ treatment partially prevented follicles loss in the ovaries of female mice treated with CTX/BUL. **(A)** Ovarian size, n = 6 mice in each group. **(C)** Representative photos of ovarian sections stained with HE, n = 3 mice. Black arrow indicates the primordial follicle; red triangle indicates the degenerative follicle. **(B)** Ovarian weight, n=6 mice. **(D)** Follicle counts in each stage, Pr, primordial follicle; Pm, primary follicles; Se, secondary follicle; Ma, mature follicle; De, degenerative follicle; PQQ, pyrroloquinoline; CTX, cyclophosphamide; BUL, busulfan. Compared with Con: *P < 0.05; **P < 0.01; ***P < 0.001; compared with O: ^#^P < 0.05.

To directly determine the ovarian reserve of mice in each group, we counted the follicles within ovaries. First, we collected the ovaries from mice and weighted the ovaries. We found that the size and weight of ovaries were significantly reduced in group O compared to group Control and group OP (**Figures 2A, B**). Compared to group Control, the size and weight of ovaries were significantly reduced in group OP (**Figures 2A, B**). Then we performed HE staining on serial ovarian sections and counted the follicles in each stage. We found that the follicle counts in each stage of ovaries were significantly reduced while the degenerative follicles were significantly increased in group O compared to group Control and group OP (**Figures 2C, D**). Compared to group Control, the follicle counts in each stage of ovaries were also significantly reduced while the degenerative follicles were significantly increased in group OP (**Figures 2C, D**). These results indicated that PQQ treatment partially protected the ovary against follicles depletion caused by CTX/BUL.

### The Effect of PQQ Treatment on Ovarian Cell Proliferation, Apoptosis and Senescence in Female Mice Treated With CTX/BUL

**Figure 3:**
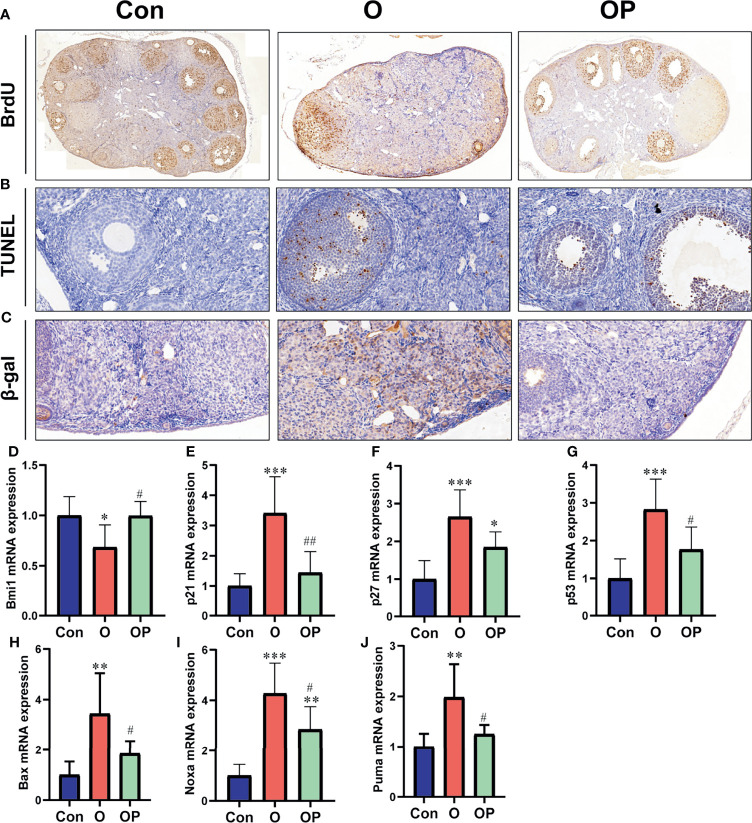
PQQ treatment promoted ovarian cell proliferation and inhibited ovarian cell apoptosis and senescence in the female mice treated with CTX/BUL. **(A)** Representative photos of ovarian sections stained with anti-BrdU antibody, n = 3 mice in each group. **(B)** Representative photos of TUNEL assay on ovarian sections, n = 3 mice in each group. **(C)** Representative photos of ovarian sections stained with anti- β-gal antibody, n = 3 mice in each group. q-PCR analysis of ovarian extracts to detect the expression of the **(D)** *Bmi1* gene, n = 6 mice in each group, **(E)** *p21* gene, n = 6 mice in each group, **(F)** *p27* gene, n = 6 mice in each group, **(G)** *p53* gene, n = 6 mice in each group, **(H)** *Bax* gene, n = 6 mice in each group, **(I)** *Noxa* gene, n = 6 mice in each group and **(J)** *puma* gene, n = 6 mice in each group. PQQ, pyrroloquinoline; CTX, cyclophosphamide; BUL, busulfan. Compared with Con: *P < 0.05; **P < 0.01; ***P < 0.001; compared with O: ^#^P < 0.05; ^##^P < 0.01.

To determine ovarian cell proliferation, apoptosis and senescence, we performed IHC and TUNEL assay on ovarian sections. We found that the proliferating cells within the ovary were granulosa cells, and the apoptosis occurred also in granulosa cells (**Figures 3A, B**). However, the senescence was evident in ovarian stromal cells (**Figure 3C**). The number of BrdU-positive cells was significantly reduced while the number of TUNEL- and β-gal-positive cells was significantly increased in group O compared to group Control and group OP (**Figures 3A–C**). To further explore molecules that may be responsible for reduced cell proliferation, increased cell senescence and apoptosis observed in group O, we detected the expression of well-known genes that play an important role in mediating cell proliferation, apoptosis and senescence. Our results showed that the gene expression of cell cycle inhibitors including *p53*, *p21* and *p27*, as well as the p53-regulated pro-apoptosis genes including *noxa, puma and bax* were significantly up regulated while the oncogene *Bmi1* was significantly down regulated in group O, compared to group Control and group OP (**Figures 3D–J**). In addition, the expression of *p27* and *noxa* were significantly up-regulated in group OP, compared to those in group Control (**Figures 3F, I**). These results indicated that PQQ treatment partially prevented the reduction of ovarian cell proliferation and increase of ovarian cell apoptosis and senescence caused by CTX/BUL.

### The Effect of PQQ Treatment on Inflammation in the Ovaries of Female Mice Treated With CTX/BUL

**Figure 4:**
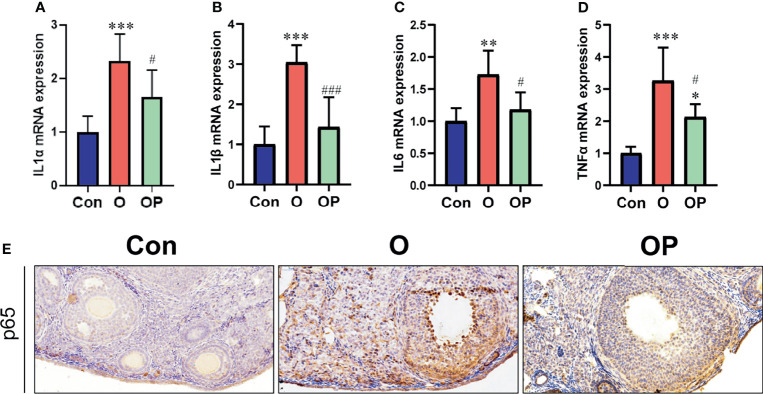
PQQ treatment decreased inflammation in the ovaries of female mice treated with CTX/BUL. q-PCR analysis of ovarian extracts to detect the expression of the **(A)** *IL-1α* gene, n = 6 mice in each group, **(B)** *IL-1β* gene, n = 6 mice in each group, **(C)** *IL-6* gene, n = 6 mice in each group, and **(D)** *TNF α* gene, n = 6 mice in each group. **(E)** Representative photos of ovarian sections stained with anti-p65 antibody, n = 3 mice in each group. PQQ, pyrroloquinoline; CTX, cyclophosphamide; BUL, busulfan. Compared with Con: *P < 0.05; **P < 0.01; ***P < 0.001; compared with O: ^#^P < 0.05; ^###^P < 0.001.

To determine the level of inflammation in ovaries in each group, first we detected the gene expression of inflammatory factors including *IL1- α, IL1-β, IL-6* and *TNF-α*. We found that the gene expression of *IL1- α, IL1-β, IL-6* and *TNF-α* was significantly up-regulated in group O, compared to group Control and group OP (**Figures 4A–D**). Compared to group Control, the gene expression of *TNF-α* was also significantly up regulated in group OP (**Figure 4D**). Then, we performed IHC on ovarian sections to detect the expression of p65 in ovaries. We found that the p65-positive cells were significantly increased in group O, compared to group Control and group OP (**Figure 4E**). These results indicated that PQQ treatment partially prevented inflammation in ovaries caused by CTX/BUL.

### The Effect of PQQ Treatment on Oxidative Damage and the Expression of Pgc1α in the Ovaries of Female Mice TreatedWith CTX/BUL

**Figure 5:**
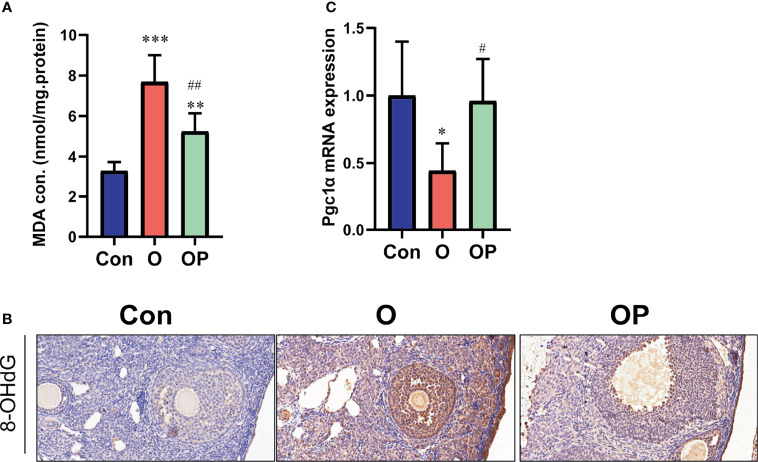
PQQ treatment decreased oxidative damage and increased the expression of *Pgc1α* in the ovaries of female mice treated with CTX/BUL. **(A)** Detection of MDA concentration from ovarian extracts, n = 6 mice in each group. **(B)** Representative photos of ovarian sections stained with anti-8-OHdG antibody, n = 3 mice in each group. **(C)** q-PCR analysis of ovarian extracts to detect the expression of the *Pgc1α* gene, n = 6 mice in each group. PQQ, pyrroloquinoline; CTX, cyclophosphamide; BUL, busulfan. Compared with Con: *P < 0.05; **P < 0.01; ***P < 0.001; compared with O: ^#^P < 0.05; ^##^P < 0.05.

To determine the effect of PQQ treatment on oxidative damage and the expression of PGC1α in the ovaries in each group, first we detected the level of MDA (a kind of lipid hydroperoxides) and 8-OHdG (a kind of oxidation products of DNA). We found that the level of MDA and the number of 8-OHdG-positive cells, were significantly increased in group O compared to group Control and group OP (**Figures 5A, B**). Compared to group Control, the level of MDA and the number of 8-OHdG-positive cells were significantly increased in group OP (**Figures 5A, B**). These results indicated that PQQ treatment partially reduced the oxidative damage in ovaries caused by CTX/BUL. Then, we detected gene expression of *PGC 1α* which plays a critical role in promoting the biogenesis of mitochondria. We showed that the gene expression of Pgc1α was significantly lower in group O than in group Control and group OP (**Figure 5C**). These results indicated that PQQ treatment partially prevented the increased oxidative damage and impaired biogenesis of mitochondria caused by CTX/BUL.

## Discussion

It is clearly demonstrated that AAs can deplete follicles in each stage within ovaries, and significantly increase the risk of POI (4). In the present study, we tested whether PQQ could protect the ovary from being damaged by CTX/BUL. We found that PQQ treatment could alleviate the loss of follicles in each stage and the increase of atretic follicles in ovaries of CTX/BUL-treated female mice and largely preserve the fertility of CTX/BUL- treated female mice.

A certain number of the primordial follicles (PMFs) which lay the foundation of female fertility exists within the ovary. Activation of PMFs initiates folliculogenesis and finally leads to ovulation. The exhausting of PMFs means the end of the reproductive lifespan. The use of AAs can significantly accelerate the loss of PMFs and the process of ovarian ageing (38). The mechanisms of AAs-induced depletion of PMFs are complicated, including the direct death of PMFs, the indirect loss of PMFs as a result of impaired microenvironment and over activation of PMFs (39). The first one may result in rapid loss of PMFs and it is not likely that this effect of AAs can be prevented by agents. Because, as anti-tumor medicines, AAs directly promote cell death by crosslinking inter-and intra-strand DNA. In contrast, the latter two may lead to gradual loss of PMFs, and provide the chance for intervention. Previous studies showed that resveratrol and stem cell transplantation could alleviate the loss of PMFs caused by AAs (11, 12). In the present study, we found that pre-treatment with PQQ also alleviated AAs-induced depletion of PMFs. We deduce that the protective effect of PQQ on preserving PMFs may rely on its effects on improving the micro-environment of PMFs and/or slowing down the rate of activation of PMFs. These still need to be further studied.

Ovarian folliculogenesis is a complicated process, filled with the communication between oocytes and granulosa cells, and supported by ovarian stroma (40). Growing follicles with activated cell proliferation of granulosa are more sensitivity to the toxicity of AAs. In the present study, the alterations of molecules in ovaries among groups were examined in 3 months after a single injection of CTX/BUL. Therefore, the alterations should not be as a result of direct toxicity of AAs but as a result of indirect toxicity of AAs. The indirect toxicity caused by AAs may have a long detrimental effects on folliculogenesis. This is demonstrated by observing an accelerated depletion of follicles even in two months after a single injection of CTX/BUL (6). One kind of the indirect toxicities caused by AAs is ROS. A high level of ROS not only damages the oocytes but attacks also ganulosa cells, leading to poor quality oocyte, even follicle atresia (41). It has been demonstrated that inhibiting the high level ROS caused by AAs could effectively improve the number of follicles in each stage (12, 42). Consistently, in the present study, we found that PQQ could significantly alleviate oxidative damage in ovaries of mice treated with CTX/BUL.

In addition to ROS, inflammation may be another kind of indirect toxicities elicited by AAs. It is known that moderate inflammation plays key physiologic roles in ovarian folliculogenesis and ovulation (43). It is clear that abnormal inflammation status in ovary may correlate with PCOS (43). Recent studies have proposed that inflammaging contributes to the development of POI (44). Said et al. have implicated that resveratrol attenuated the radiation induced POI by inhibiting inflammatory signaling (45). Consistently, Jiang et al. found that resveratrol improved the follicle counts of mice treated with CTX/BUL, at least partly by lowering the level of inflammation in ovary (12). It is well demonstrated that PQQ plays a critical role in inhibiting the inflammatory response, and the inflammation-inhibitory effect is associated with inactivation of the p65 signaling pathway (46, 47). As expected, in the present study, we found that PQQ down-regulated the gene expression of pro-inflammatory factors in ovary and inactivated the expression of p65 in ovarian stromal cells. It has been demonstrated that numerous kinds of immune cells present in ovarian stroma (48). It is possible that these immune cells may contribute to the abnormal inflammation in ovary of mice treated with CTX/BUL, and PQQ may exert a role in regulating these cells. However, these need to be further studied.

Mitochondria called energy factory is important for somatic cells, and particular important for oocytes. Optimal mitochondrial function is required for oocyte maturation, fertilization, and embryonic development (49). It has been demonstrated the feasibility of mitochondrial replacement therapy for treatment of infertility (50). Mitochondria is a target of AAs, and AAs-induced ROS may further damage the intracellular mitochondria (51). A previous study showed a protective effect of Crocetin and AS101 against the ovarian damage caused by CTX partly by increasing the biogenesis of mitochondria (13). PQQ is known as a potent stimulator of mitochondrial genesis (34). As expected, in the present study, we found that PQQ treatment increased the gene expression of *Pgc 1α* which plays a critical role in promoting mitochondrial biogenesis, indicating that PQQ treatment may promote the biogenesis of mitochondria.

Indirect toxicities of AAs including ROS, inflammation and mitochondrial damage may result in chronic stress that directly damage the process of folliculogenesis. In the present study, the number of BrdU positive cells was reduced significantly in ovaries of CTX/BUL-treated mice, however, the remaining growth follicles in the ovaries of CTX/BUL-treated mice had normally proliferating granulosa cells, indicating a reduced number of growth follicles in the ovaries of CTX/BUL-treated mice. We found that the number of apoptotic granulosa cells and the expression of genes promoting cell apoptosis were increased in ovaries of CTX/BUL-treated mice. A recent study conducted by Victor et al. indicated that cellular hallmarks of aging emerge in the ovary prior to primordial follicle depletion (52). In that study, they observed a significantly increased expression of genes related to pro-inflammatory stress and cell-cycle inhibition in naturally aged ovaries of mice. Consistently, in the present study, we also observed an increased number of senescent stromal cells and an increased expression of genes related to cell-cycle inhibition in the ovaries of CTX/BUL-treated mice. As expected, PQQ pre-treatment attenuated granulosa apoptosis and stromal cell senescence in the ovaries of CTX/BUL-treated mice. Therefore, we concluded that PQQ may attenuate the indirect toxicities caused by AAs including oxidative stress, inflammation and mitochondrial damage, thereby protect the follicles in each stage from being damaged, finally preserve the fertility of mice.

Oxidative stress, poor mitochondrial genesis and inflammation are also involved in the process of naturally ovarian aging (41, 53, 54). Therefore, we deduced that PQQ may exert a protective effect against natural ovarian aging. This will be investigated in our future study. As we mentioned before, AAs are used for treatment of cancers. A concern is whether PQQ may promote the growth and migration of cancer cells. Although, a previous study has demonstrated an anti-tumor effect of PQQ on *in vitro* cultural cancer cell lines (55). It is possible that PQQ protects both normal and cancer cells. Therefore, before excluding a non-tumor promoting effect of PQQ, PQQ should not be advocated for the purpose of fertility preservation in women with cancer.

## Conclusion

In conclusion, our study demonstrated a role of PQQ in improving ovarian function following damage by AAs, suggesting that PQQ could be potentially used as an agent for preventing ovarian aging and preserving fertility.

## Data Availability Statement

The original contributions presented in the study are included in the article/**Supplementary Material**. Further inquiries can be directed to the corresponding authors.

## Ethics Statement

The animal study was reviewed and approved by The Institutional Animal Care and Use committee of Nanjing Medical University and the Ethics Committee of Changzhou Maternal and Child Health Care Hospital.

## Author Contributions

Substantial contribution to conception and design: LC, DM, and JW. The experiments and data acquisition were performed by XD, XY, YW, WX, and JT. Data analysis: XD, XY, and YW. Data interpretation: all authors. Drafting the article: XD and XY. Critical revision of the article for important intellectual content: all authors. All authors contributed to the article and approved the submitted version.

## Funding

This project was supported by the National Natural Science Foundation of China (No.81901436) to XD, Changzhou Health Committee Funded Young Investigator Training Project (CZQM2020094) to XD. Program of Jiangsu Province’s Key Provincial Talents of Women and Child Health Care (FRC 201751) and Key Program of Changzhou Municipal Health Commission (ZD201921) to LC.

## Author Disclaimer

The views and opinions described in this publication do not necessarily reflect those of the grantor.

## Conflict of Interest

The authors declare that the research was conducted in the absence of any commercial or financial relationships that could be construed as a potential conflict of interest.

## Publisher’s Note

All claims expressed in this article are solely those of the authors and do not necessarily represent those of their affiliated organizations, or those of the publisher, the editors and the reviewers. Any product that may be evaluated in this article, or claim that may be made by its manufacturer, is not guaranteed or endorsed by the publisher.
