# Supplementary material for: PQQ Dietary Supplementation Prevents Alkylating Agent-Induced Ovarian Dysfunction in Mice
Source: Front Endocrinol (Lausanne). 2022 Mar 7;13:781404. doi: 10.3389/fendo.2022.781404 (PMC8948422; doi:10.3389/fendo.2022.781404)
Supplement: Supplementary file 1 [file Image_1.pdf]

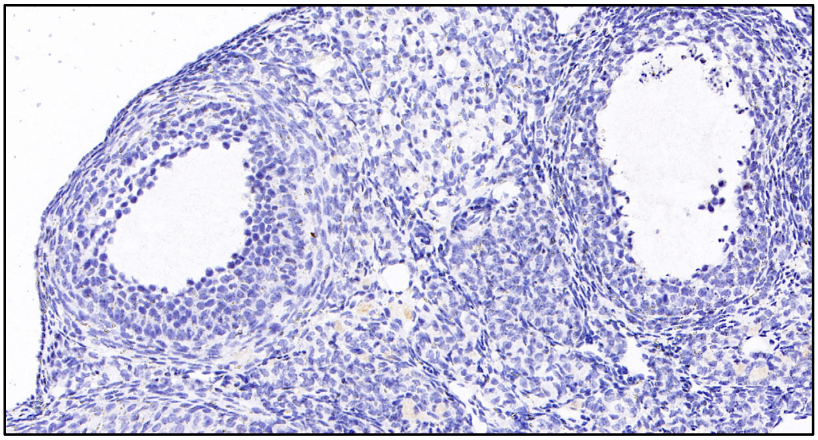

Supplementary figure 1 An image of negative control for IHC. Ovarian section without incubating with primary antibody.
