# Supplementary material for: PQQ Dietary Supplementation Prevents Alkylating Agent-Induced Ovarian Dysfunction in Mice
Source: Front Endocrinol (Lausanne). 2022 Mar 7;13:781404. doi: 10.3389/fendo.2022.781404 (PMC8948422; doi:10.3389/fendo.2022.781404)
Supplement: Supplementary file 2 [file Table_1.docx]

| Name | S/AS | Sequence | Tm (℃) | | Length (bp) | |
| --- | --- | --- | --- | --- | --- | --- |
| Bmi1  p53  p21  p27  Bax  Noxa  Puma  IL1α  IL1β  IL6  TNFα  PGC1α | S  AS  S  AS  S  AS  S  AS  S  AS  S  AS  S  AS  S  AS  S  AS  S  AS  S  AS  S  AS | 5’- ATTGATGCCACTACCATAAT-3’  5’- CCTGGACATCACAAATAGGAC-3’  5’- GACCGCCGTACAGAAGAAGAAA-3’  5’- CGGAACATCTCGAAGCGTTTAC-3’  5’- GTCTTGCACTCTGGTGTCTGAG-3’  5’- TGCGCTTGGAGTGATAGAAA-3’  5’- GATGTCAAACGTGAGAGTGTCTA-3’  5’- CTGACTCGCTTCTTCCATATCC-3’  5’- CGAGCTGATCAGAACCATCAT-3’  5’- GCCACAAAGATGGTCACTGT-3’  5’- GAACGCGCCAGTGAACCCAA -3’  5’- CTTTGTCTCCAATCCTCCGG -3’  5’- CTGGAGGGTCATGTACAATCTCTT-3’  5’- CACCTAGTTGGGCTCCATTTCT-3’  5’ TGGTTAAATGACCTGCAACAGGAA-3’  5’- AGGTCGGTCTCACTACCTGTGATG-3’  5’- TCCAGGATGAGGACATGAGCAC -3’  5’- GAACGTCACACACCAGCAGGTTA -3’  5’- GTTCTCTGGGAAATCGTGGA -3’  5’- GGAAATTGGGGTAGGAAGGA -3’  5’- TATGGCCCAGACCCTCACA -3’  5’- GGAGTAGACAAGGTACAACCCATC -3’  5’- CGGAAATCATATCCA ACCAG -3’  5’- TGAGGACCGCTAGCA AGTTTG -3’ | | 58  55  55  60  60  60  60  60  61  62  62  62 | | 128  105  202  185  134  250  117  120  272  96  221  215 |

**Supplementary Table 1** The primers of genes detected in the present study

S: sense primer; AS: anti-sense primer.
